# Spinal neuronal excitability and neuroinflammation in a model of chemotherapeutic neuropathic pain: targeting the resolution pathways

**DOI:** 10.1186/s12974-020-01997-w

**Published:** 2020-10-23

**Authors:** Pongsatorn Meesawatsom, Gareth Hathway, Andrew Bennett, Dumitru Constantin-Teodosiu, Victoria Chapman

**Affiliations:** 1 Pain Centre Versus Arthritis, School of Life Sciences, Medical School, University of Nottingham, Nottingham, NG7 2UH UK; 2 Department of Pharmacology, Faculty of Pharmacy, Mahidol University, Rajathevi, Bangkok, 10400 Thailand; 3 FRAME Alternatives Laboratory, School of Life Sciences, Medical School, University of Nottingham, Nottingham, NG7 2UH UK; 4 MRC/ARUK Centre for Musculoskeletal Ageing Research, School of Life Sciences, Medical School, University of Nottingham, Nottingham, NG7 2UH UK

**Keywords:** AT-RvD1, Resolvins, Pain, Inflammation, Neuropathy, Chemotherapy, Paclitaxel, Electrophysiology, Pathway analysis

## Abstract

**Background:**

Neuroinflammation is a critical feature of sensitisation of spinal nociceptive processing in chronic pain states. We hypothesised that the resolvin pathways, a unique endogenous control system, may ameliorate aberrant spinal processing of somatosensory inputs associated with chemotherapy-induced neuropathic pain (CINP).

**Method:**

The paclitaxel (PCX) model of CINP was established in male Sprague-Dawley rats and compared to control rats (*n* = 23 and 22, respectively). Behavioural pain responses were measured, and either single unit electrophysiological recordings of dorsal horn wide dynamic range (WDR) neurones were performed, or mRNA microarray analysis of the dorsal horn of the spinal cord was undertaken.

**Results:**

PCX rats exhibited significant changes in behavioural responses to mechanical and cold stimuli. A higher proportion of WDR neurones in PCX rats were polymodal (generating post-discharge following a non-noxious mechanical stimulus, responding to non-noxious cold and exhibiting spontaneous activity) compared to control (*p* < 0.05). Microarray analysis revealed changes in proinflammatory pathways (*Tlr*, *Tnfrsf1a*, *Nlrp1a*, *Cxcr1*, *Cxcr5*, *Ccr1*, *Cx3cr1*) and anti-inflammatory lipid resolvin pathways (*Alox5ap*, *Cyp2j4* and *Ptgr1*) compared to control (*p* < 0.05). Ingenuity pathway analysis predicted changes in glutamatergic and astrocyte signaling in the PCX group. Activation of the resolvin system via the spinal administration of aspirin-triggered resolvin D1 (AT-RvD1) markedly inhibited (73 ± 7% inhibition) normally non-noxious mechanically (8 g) evoked responses of WDR neurones only in PCX rats, whilst leaving responses to noxious mechanically induced stimuli intact. Inhibitory effects of AT-RvD1were comparable in magnitude to spinal morphine (84 ± 4% inhibition).

**Conclusion:**

The PCX model of CINP was associated with mechanical allodynia, altered neuronal responses and dysregulation of pro- and anti-inflammatory signalling in the spinal dorsal horn. The resolvin AT-RvD1 selectively inhibited low weight mechanical-evoked responses of WDR neurones in PCX rats, but not in controls. Our data support the targeting of spinal neuroinflammation via the activation of the resolvin system as a new therapeutic approach for CINP.

## Introduction

Chemotherapy-induced neuropathic pain (CINP) is a common adverse effect of antineoplastic agents, limiting the tolerability of cancer treatment. Paclitaxel (PCX), also known as taxol, is associated with a high estimated prevalence of CINP (70%) as assessed at 1-6 months after treatment [1]. PCX is a tubulin-stabilising factor that inhibits tumour cell proliferation, but which also affects neuronal function by disrupting axonal transport and causes demyelination leading to length-dependent neuropathy [2]. PCX-induced CINP manifests predominantly with sensory neuropathy symptoms including numbness, burning and allodynia upon mechanical and cold stimuli [3].

Experimental models of PCX-induced CINP in rats are associated with pain behaviour [4] and broad changes in the peripheral and central nervous system, including altered PGP9.5-positive intra-epidermal nerve endings [5], peripheral nerve mitochondrial morphology and mitochondrial bioenergetics of dorsal root ganglia (DRG) neurones [6]. Activation of toll-like receptor 4 (TLR4) plays important roles in both DRG and the spinal cord [7], with down-stream increased expression of pro-inflammatory cytokines and chemokines in DRGs and marked increases in numbers of DRG macrophages [8, 9].

Spinal plasticity associated with PCX-treatment includes changes in N-methyl-D-aspartate (NMDA) receptors, the calcium channel subunit α2δ1 [10], γ-aminobutyric acid and glutamate transporters [11, 12], which all promote spinal hyperexcitability. Activation of spinal astrocytes in the PCX-model is rapid and sustained, and associated with a downregulation of glutamate transporters [13]. Increased expression of several cytokines and chemokines [14], which are released from microglia, astrocytes and neurones, is widely acknowledged to be central to neuroinflammatory processes associated with pain [15, 16].

Specialised pro-resolving mediators (SPMs) are a diverse family of potent-anti-inflammatory lipids including the D-series resolvins, resolvin D1 (RvD1) and its more enzymatic resistant isomer, aspirin-triggered-RvD1 (AT-RvD1) [17], which are generated after an overt inflammatory insult to promote resolution [18, 19]. Effects of the resolvins include inhibition of pro-inflammatory cytokine production and the shortening of the inflammation-resolution interval by inhibiting neutrophil infiltration and stimulating macrophage phagocytosis [19]. Spinal administration of resolvin E1 (RvE1) prevents and reverses neuropathic pain behaviour [20] and spinal RvD1 attenuated mechanical hypersensitivity in PCX-treated mice [21]. Sexual dimorphism was reported for some of the resolvins in this previous study, specifically for RvD5, but not RvD1 or RvD2-mediated analgesia in the PCX model [21]. Previously, we reported that spinal administration of AT-RvD1 has robust inhibitory effects on noxious-evoked responses of spinal neurones in a model of acute inflammatory pain, but does not alter somatosensory processing in the spinal cord in naïve rats [22]. Here, we hypothesised that the sensitisation of spinal neuronal activity in the PCX model would be associated with changes in the spinal expression of genes encoding key neuroinflammatory markers and enzymes involved in the generation/catabolism of the resolvins, and that PCX-induced changes in spinal neuronal excitability would be responsive to spinal treatment with AT-RvD1.

## Materials and methods

### Animals

Male Sprague-Dawley rats (*n* = 58) were purchased from Charles River, UK (weight range at the start of studies 175-250 g). The studies, which were carried out in accordance with the UK Home Office Animals (Scientific Procedures) Act (1986) and followed the guidelines of the International Association for the Study of Pain [23], were approved by the local ethical review board at the University of Nottingham. Rats were group housed at the Bio Support Unit, University of Nottingham, in open cages with food and water available ad libitum. In accordance with the Animal Research: Reporting of In Vivo Experiments (ARRIVE) guidelines [24], full details of the group sizes for the different studies and experimental endpoints are in Supplemental file: Table 1.

### Induction of pain models

#### Paclitaxel-induced CINP model

Rats received intraperitoneal injection of paclitaxel (PCX; Tocris, UK) 2 mg/kg (*n* = 11) or vehicle control (10% Cremophor, 5% ethanol in saline) (*n* = 9) on 4 alternate days (cumulative dose of 8 mg/kg) [4]. Rats were randomly divided into four groups for 2 separate studies including electrophysiological studies (PCX, *n* = 16 and vehicle, *n* = 15) and gene expression studies (PCX and vehicle, *n* = 7 each).

#### Carrageenan-induced paw inflammation

As a comparator for gene expression study, a model of acute inflammation was also studied. Under brief anaesthesia (isoflurane 2.5–3% in O_2_ at 1 l/min flow), rats received either an intraplantar injection of 2% λ-carrageenan 100 μl (Sigma, UK) (*n* = 6) or vehicle (0.9% saline; *n* = 7) into the glabrous surface of the left hindpaw [25].

### Pain behaviour measurement

Pain behaviour was quantified as previously described [26] in a blinded fashion at baseline, 2, 8 and 24 h post-carrageenan injection and twice a week post PCX injection for 28 days. For the carrageenan model, weight-bearing asymmetry, reflecting the change in weight bearing from the ipsilateral hind limb to the contralateral hind limb, was measured using an incapacitance tester (Linton Instrumentation, UK). For both carrageenan and PCX model, paw withdrawal thresholds (PWTs) of both hindpaws were assessed using the up-down method [27] with von Frey monofilaments with a range of bending forces (0.6, 1, 1.4, 2, 4, 6, 8, 10, 15 and 26 g), starting at 4 g. Once a withdrawal reflex was observed, the next descending monofilament was applied to retest until no response was elicited. PWT was determined as the lowest force of monofilament, which evoked a paw withdrawal reflex [28]. For the PCX model, acetone [29] evoked nocifensive duration and paw withdrawal latency were assessed once a week, always following a PWT test. Acetone was applied by a single droplet (~ 100 μl) using a polystyrene tubing attached to a 5 ml syringe. The test was carried out alternately on each hindpaw three times, with 3-5 min interval between each test. Nocifensive behaviour, including brisk foot withdrawal, flinching, shaking and licking the plantar surface of the foot was observed and timed. The total duration of nocifensive behaviours in responses to acetone applications (3 times/paw) was calculated. Rats received 1-2 sessions of acetone test habituation before the experiment to minimise any confounding effect of acetone odour. The overall gain in body weight and general behaviour of the rats was monitored throughout the study. None of rats developed signs and symptoms of severe distress, abnormal weight gain or weight loss or death during behavioural experiments.

### In vivo spinal electrophysiology

Single-unit in vivo electrophysiology recording of dorsal horn wide dynamic range (WDR) neurones was performed, as previously described [30] on days 28–32 following induction of the PCX model and in the vehicle-injected control groups. At this timepoint, PCX-injected rats developed robust mechanical and cold hypersensitivity which coincides with the reduced PGP9.5 positive intraepidermal nerve fibre density, a maker of peripheral neuropathy [5, 31–35].

Rats were anaesthetised with isoflurane (3% induction, 2% surgery, 1.50-1.75% maintenance) in 0.6 l/min N_2_O and 0.3 l/min O_2_, and a tracheal cannula was inserted. Rats were placed in a stereotaxic frame, and a laminectomy was performed to expose the L4–L5 region of the spinal cord receiving the input from the hindpaw. The spinal column was held rigidly by clamps caudal and rostral to the exposed section. Extracellular single-unit recordings of deep (500–1000 μm, laminae V-VI) wide dynamic range (WDR) dorsal horn neurones were made with glass-coated tungsten microelectrodes. Laminar V–VI WDR neurones have well-characterised responses to noxious stimuli and exhibit graded responses to noxious stimuli. Responses of WDR neurones following natural (mechanical and cold) and artificial (electrical) stimuli delivered at the centre of the receptive field on the hindpaw were characterised. The electrical activity of spinal neurones was amplified, filtered by a Neurolog system (Digitimer, UK), digitised by CED Micro1401 (Cambridge Electronic Design, Cambridge, UK) and captured/analysed by the Spike 2 version 6.05 software (Cambridge Electronic Design, UK). Trains of 4 von Frey filaments (8, 10, 15 and 26 g) were applied for 10 s with a 10-s interval between each filament followed by a drop of acetone (~ 100 μl) 10-30 s after mechanical stimulation. Following an interval of 5 min, a train of 16 (0.5 Hz, 2-ms pulse width) consecutive electrical stimulations at three times (3×) the threshold for C-fibres and then 3× the threshold for Aβ fibres was delivered to the hindpaw via transcutaneous electrodes. Cycles of stimuli were carried out in 15-min intervals. The neuronal action potentials in spikes/s evoked by mechanical and cold stimuli were recorded using rate metre in the Spike 2 software. The number of action potentials (APs) evoked by electrical stimulus was quantified on the basis of latencies as Aβ (0-20 ms), Aδ (20-90 ms), C-fibre (90-300 ms), post-discharge (PD, 300-800 ms), non-potentiated responses or input (C-fibre response from the first electrical stimulus multiply by 16) and potentiated responses or wind-up (WU, total APs in 90-800 ms latency minus input) as previously described [22, 30]. Core body temperature was maintained (36.5–37.0 °C) via a homeothermic heated blanket linked to a rectal probe (Harvard Instruments, UK).

### Pharmacological studies

Following stable control-evoked neuronal responses (< 10% variation of C-fibre and natural stimulus responses), drugs were applied to the surface of the exposed L4-5 segments of the spinal cord via a Hamilton syringe in 50-μl volume. The concentrations of AT-RvD1 were based on previous studies [22, 36]. PCX and vehicle control rats received spinal AT-RvD1 (Cayman Chemical, USA) cumulatively (15 ng and 150 ng /50 μl) and then spinal morphine sulphate (Queen’s Medical Centre Pharmacy, Nottingham, UK) 1 μg/50 μl in PBS vehicle, which has been previously shown to produce a 60-75% inhibition in a model of neuropathic pain [37], and was therefore chosen as the positive control. Effects of treatments on evoked responses of the neurones were followed for 60 min post-treatment.

### TaqMan low-density array for spinal gene expression quantification

In separate groups of rats, following the last time point of pain behaviour assessment (30 h post-carrageenan or 28 days post-PCX), rats were sacrificed by cranial concussion and decapitated. This timepoint in the carrageenan model was based on our previous report of changes in the resolvin system in the dorsal horn of the spinal cord [22]. The lumbar enlargement (L4-L6) of the spinal cord was dissected free, and the ipsilateral dorsal horn quadrant was frozen on dry ice and stored at −80 °C until use. After treating with 2 ml ice-cold Tri-reagent (Sigma Aldridge, UK), total RNA was extracted and isolated from the tissue samples according to the manufacturer’s instructions. Complementary DNA (cDNA) was synthesised by reverse transcription from 500 ng total RNA using SuperScript III (Invitrogen, UK) reverse transcriptase according to the manufacturer’s instructions. Reactions were incubated at 25 °C for 10 min, 37 °C for 50 min and followed by 70 °C for 15 min to complete the reaction. Multiple mRNA expression measurements were made according to the manufacturer’s instructions on 100 ng of cDNA obtained from total mRNA isolated from spinal cord tissue collected from animals in each treatment group using Applied Biosystems 384-well microfluidics TaqMan array cards (96 gene format). Genes investigated were selected according to a search of the literature, and SA Biosciences and IPA databases. They were grouped into 8 clusters, (1) resolvin systems, (2) pro-inflammatory cascades, (3) anti-inflammatory cascades, (4) glial markers, (5) markers for central sensitisation, (6) chemokines (7) chemokine receptors and (8) reference genes. The complete list of genes, synonyms, protein products and ABI assay IDs are shown in Supplemental file: Table 2. To quantify mRNA expression, the sealed cards were loaded into ABI PRISM 7900 HT Sequence Detection System (Applied Biosystems, USA) controlled by the Sequence Detection System (SDS) software version 2.1. Cards were incubated with the following PCR cycling conditions: 2 min at 50 °C, 10 min at 95 °C and 40 cycles of 30 s at 97 °C and 1 min at 59.7 °C. From the profile of the fluorescence curves, the cycle threshold (Ct) values were calculated using RQ Manager 1.2.2. Expression stability of candidate reference genes was calculated by a Visual Basic Application (VBA) for Microsoft Excel—termed geNorm [38] and expressed as *M* values, which are inversely related to the stability. β-actin (*Actb*) and mitogen-activated protein kinase 6 (*MAPK6*) had the lowest *M* value serving as suitable reference genes.

### Statistical analysis of physiological and gene card data

GraphPad Prism 7.0 (GPP 7.0; GraphPad Software, Inc, San Diego, CA) was used for statistical analyses and graphical presentation. Data were excluded from the statistical analysis, where outliers were identified by Grubb’s test at *α* = 0.05 which excludes only 1 outlier from the group. Results are expressed as either the median ± interquatile range or mean ± standard deviation (SD) depending on the data distribution. Kolmogorov-Smirnov test was used to determine whether data are normally distributed. Percentage of weight (wt) bearing asymmetry was calculated from the formula [(Contralateral wt − Ipsilateral wt) / (Contralateral wt + Ipsilateral wt)] × 100. Pain behaviour data were analysed with a two-way analysis of variance (ANOVA) with Sidak post hoc test.

When analysing correlations between evoked responses of spinal WDR neurones and pain behaviour, if > 1 neurone from an animal was characterised, the evoked response from those neurones was averaged. Correlations of pain behaviour and evoked responses were performed using two-tailed Spearman’s rank test. Differences in the proportion of neurones between PCX and control were analysed using Fisher’s exact tests. Evoked responses of the spinal neurones following spinal application of AT-RvD1 (15 and 150 ng/50 μl) were compared to baseline using repeated-measures ANOVA followed by Sidak post hoc test or Friedman statistics followed by Dunn’s post hoc test. Percentages of maximal inhibition were calculated versus baseline (pre-drug responses) and were compared between groups using the Kruskal-Wallis test with Dunn’s post hoc test. Evoked responses following spinal morphine 1 μg/50 μl application were compared to baseline using paired *t* tests or nonparametric Wilcoxon tests. Statistical analysis of gene expression data was performed using a Mann-Whitney *U* test with Bonferroni correction.

To correlate the pain behaviour and the levels of spinal gene expression, the absolute PWT on day 28 was transformed to the number of von Frey filament changed from baseline (ΔvF), for example baseline PWT = 26, day28 PWT = 4, ΔvF = 5. Statistical significance was considered where the *p* value is ≤ 0.05 for all comparisons.

### Bioinformatic analysis

The Ct values generated by the Applied Biosystems RQ Manager software were normalised to the pooled geometric mean of *Actb* and *MAPK6* genes [39]. For individual mRNA expression data, the fold-change difference in gene expression in intervention groups relative to saline was calculated with the 2^−ΔΔCt^ method with saline group serving as control/calibrator, before being uploaded in the ingenuity pathway analysis (IPA) software (Qiagen, Hilden, Germany) for pathway analysis of gene expression data. Data analysis and interpretation with IPA builds on the comprehensive, manually curated content of the ingenuity knowledge base. Algorithms identify regulators, relationships, mechanisms, functions, and pathways relevant to changes observed in an analysed dataset. The IPA analysis of the array was focused on the 96 selected genes, rather than an unbiased analysis of gene expression at a global level.

*R* was used to generate the heat map, which depicts the Z-score normalisation of the log_2_ of the fold-change gene expression normalised to calibrator (control groups), which were calculated as: *x*[*i*,*j*] z-score-normalised = (*x*[*i*,*j*]−mean[i])/stdv[*i*]. Data in each column are centred on zero, as the mean across all time points and interventions was subtracted from all values, and the results were divided by the standard deviation, to prevent those rows with little variation losing contrast.

Differences in cellular functions from control associated with interventions were expressed as *p* values generated by IPA of dorsal horn tissue mRNA expression data generated using microfluidic TaqMan array cards. The *p* value associated with each cellular function was a measurement of the likelihood that the association between a set of relevant up- or downregulated transcripts and a given function was due to random chance. The -log of *p* value was calculated using Fisher’s exact test (right tailed). A function was considered statistically significantly activated (or inhibited) with an overlap *p* value ≤ 0.05 and an IPA activation Z-score ≥ 2.0 (or ≤ −2.0). The overall outcome of each IPA analysis (e.g., upstream regular analysis, cellular function, activation status) was predicted by calculating a regulation Z-score and an overlap *p* value, which were based on the number of regulated target genes of an interest function, size changes and direction of expression of these genes, and their agreement with the IPA database constructed on curated literature searches. Correlations between mRNA expression of genes for the resolvin system and significant genes identified from either 2^−ΔΔCt^ method or IPA analysis were performed using two-tailed Spearman’s rank test.

## Results

### Altered spinal neuronal response profiles in the paclitaxel model of chemotherapy-induced neuropathic pain

**Fig. 1:**
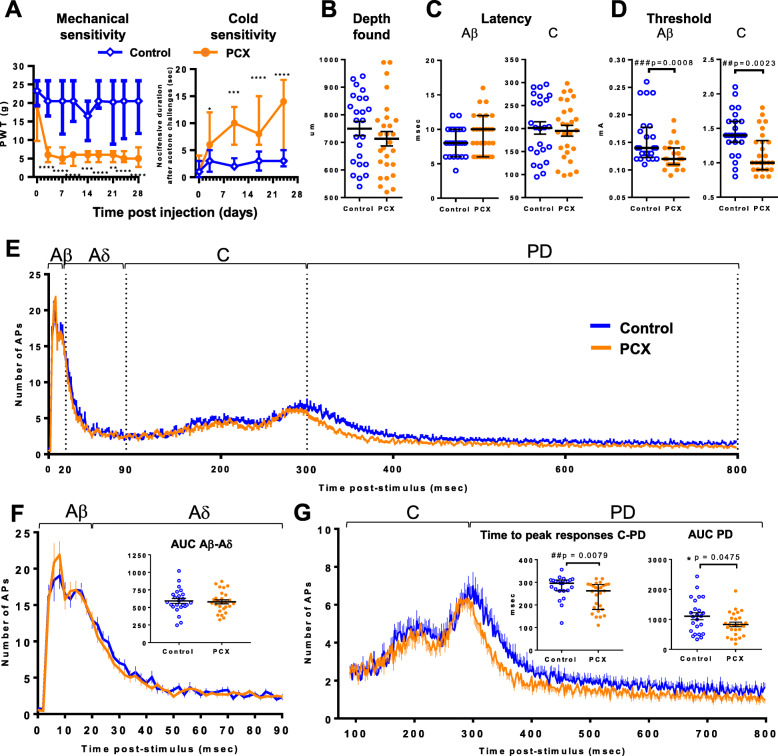
PCX model: responses of spinal WDR neurones. (**a**) Following intraperitoneal injection of PCX (2 mg/kg on four alternate days (*n* = 16)) rats had significantly decreased paw withdrawal thresholds and increased nocifensive duration after acetone challenge, compared to control treatment (10% cremophor, 5% ethanol in the saline vehicle, *n* = 15 rats). *p < 0.05, **p < 0.01, ****p < 0.001, ****p < 0.0001, Two-way repeated measure ANOVA with Sidak’s post hoc test. (**b-f**) On day 28, PCX and vehicle rats were prepared for the electrophysiology recording of spinal wide dynamic range neurones. Recorded WDR neurones from PCX (*n* = 27 neurones) and control (*n* = 24 neurones) groups had similar depths in the dorsal horn (**b**) and similar Aβ and C-fibre latencies (**c**). Thresholds for Aβ and C-fibre evoked responses were significantly lower in the PCX group, compared to control (**d**). (**e**) Post-stimulus histograms (0-800 ms) of the responses of WDR neurones following a train of 16 electrical stimuli (at 3× C-fibre threshold, recorded in 2 ms bins). (**f**) There were no differences in the magnitude of responses of neurones in the Aβ and Aδ latency band (0-90 ms) between the two groups of rats. (**g**) There was an earlier time to peak response in C-fibre and PD latency band (90-800 ms) in PCX rats, compared to control. There was a significantly smaller neuronal response in the PD latency in the PCX rats (insert). Analysis with unpaired *t* test: *p < 0.05 and Mann-Whitney *U* test: #*p* < 0.05, ##*p* < 0.01. Note that > 1 neurone was characterised per rat. Most data expressed as median ± interquatile range except AUC PD in (**g**) which data were normally distributed

Prior to electrophysiological characterisation, PCX-treated rats (*n* = 16) exhibited marked mechanical hypersensitivity and cold allodynia evident by a significant lowering of PWT (*F* (df = 8, 232) = 4.129, *p* < 0.001, two-way ANOVA) and higher acetone-induced nocifensive behaviour duration (*F* (df = 4, 116) = 5.491, *p* < 0.001, two-way ANOVA), from day 3 after the first dose of PCX onwards, compared to control rats (*n* = 16; Fig. 1a). Spinal WDR neurones (*n* = 51, depths corresponding to laminae V–VI of the dorsal horn, Fig. 1b) were characterised under anaesthesia 28-32 days following model induction (PCX, *n* = 16 and vehicle control, *n* = 15).

Latencies of the Aβ- and C-fibre evoked responses were similar across groups (Fig. 1c); however, electrical thresholds for Aβ and C-fibre activation of WDR neurones in the PCX rats were significantly lower than control (Fig. 1d, all *p* < 0.01, Mann-Whitney *U* tests). The number of APs evoked by electrical stimulation was plotted as a post-stimulus-time histogram of the neuronal responses from 0-800 ms latency post electrical stimulation is shown in Fig. 1e. The number of APs was overall similar (Supplemental file: Table 3), except during the post-discharge period when numbers of action potentials in PCX rats were significantly lower than in controls (*p* < 0.047, unpaired *t* test). AUC analysis of the PSTH confirmed that responses in PD latency bands in the PCX rats were significantly smaller than in controls (*p* < 0.047, unpaired *t* test, Fig. 1g right inset). Interestingly, the latency-to-peak response in the C-fibre/PD band in the PCX group was significantly earlier than in control rats (*p* < 0.0079, Mann-Whitney *U* test, Fig. 1g left inset). Hindpaw mechanical evoked responses in the PCX group tended to be smaller than in controls, but significance level was only reached for the 15 g evoked response (26 ± 2 APs/s versus 47 ± 6 APs/s, respectively, *p* = 0.025 Mann-Whitney *U* test, Table S2).

**Fig. 2:**
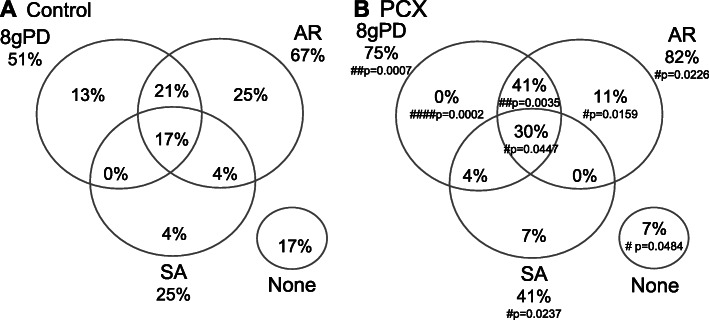
Comparison of response properties of WDR neurones in PCX and control groups. WDR neurones were classified by their response profile: post-discharge following 8 g von Frey hair stimulation (8 gPD); acetone responsive (AR); spontaneous activity (SA). WDR neurones in the PCX group exhibited greater polymodality than the control group. Analysis with Fisher’s exact test: #*p* < 0.05, ###*p* < 0.01, #####*p* < 0.0001. Control, *n* = 24 and PCX, *n* = 27 neurones

We then asked whether there was evidence of a change in the proportion of WDR neurones displaying characteristics of hyperexcitability (spontaneous activity (SA); generation of post-discharge after low-intensity mechanical stimulation by 8 von Frey hair (8gPD) and acetone responsiveness (AR)) in the PCX model (Fig. 2). A more significant proportion of WDR neurones in PCX rats were responsive to either 8gPD, AR or SA compared to controls. The proportion of neurones exhibiting multiple combinations of properties, 8gPD + AR or 8gPD + AR + SA, were significantly higher in PCX rats than in controls (41% versus 21% (*p* = 0.0035, Fisher’s exact test) and 30% versus 17% (*p* = 0.0447, Fisher’s exact test), respectively, Fig. 2). Conversely, the proportion of neurones lacking all of these properties in the PCX rats was significantly lower than in controls (*p* < 0.0484, Fisher’s exact test; Fig. 2). Thirteen percent of the recorded neurones in control rats exhibited only the 8gPD characteristic, whereas none of the recorded neurones in the PCX group exhibited this characteristic alone (*p* < 0.0002, Fisher’s exact test). Collectively, these data suggest that PCX treatment significantly changed the ongoing and evoked responses of WDR neurones in the deep dorsal horn, so that they exhibited increased polymodal sensitivity, which is a characteristic of clinical CINP.

### Dynamic changes in spinal gene expression in the PCX model support central sensitisation mechanisms

Next, we assessed whether the changes in WDR neuron response characteristics in the PCX model were associated with altered neuroinflammatory responses in the PCX group. Using ipsilateral dorsal horn spinal cord quadrants from time-matched groups of rats (28-day post-PCX model versus vehicle control), TaqMan® Low-Density Array (TLDA) analysis of 91 target genes (plus five reference genes) was performed (Supplemental file: Table 2). In order to observe distinct pattern of neuroinfammation amongst different types of pain, a second model of acute pain (intraplantar injection of carrageenan) was also included as a comparator; short-term model of hindpaw inflammation. Pain behaviour for all groups of rats is shown in Supplemental file: Fig. 1.

**Fig. 3:**
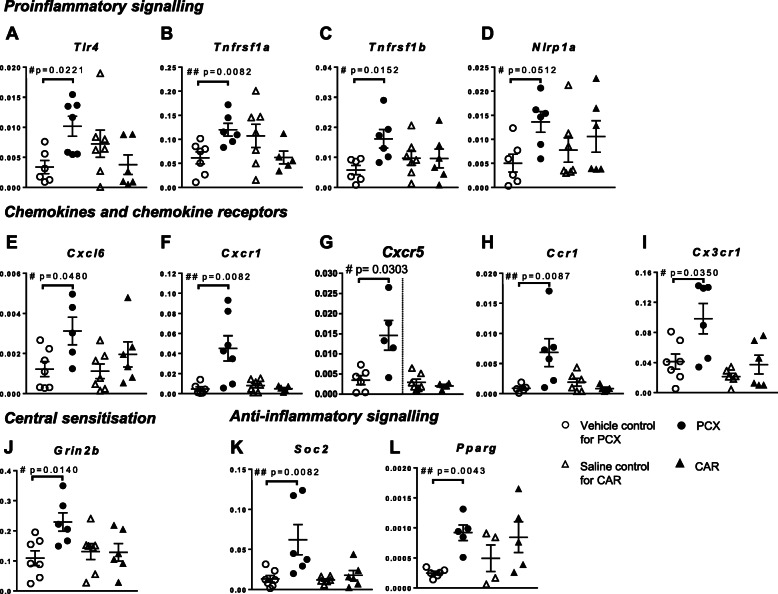
Spinal mRNA expression of selected genes in the PCX and carrageenan models versus their controls. mRNAs were collected from the ipsilateral dorsal quadrant of the lumbar enlargement region (lumbar 4-6 of the) spinal cord. Data expressed as median ± interquatile range of Δ^CT^ of PCX (filled circles) and carrageenan (filled triangles) compared to respective controls (opened symbols). Analysis with Mann-Whitney *U* test: #*p* < 0.05, ##*p* < 0.01 versus corresponding saline/vehicle, *n* = 5-7 rats in each group

Transcript levels of pro-inflammatory signalling molecules, TLR receptor-4 (*Tlr4*), the NLRP1A inflammasome (*Nlrp1a*) and TNF-α receptors (*Tnfrsf1A* and *Tnfrsf1B*) in the PCX group were significantly higher than in the control group (*p* = 0.05, Mann-Whitney *U* test, fold change (FC) = 2.0-2.9) (Fig. 3a-d). Levels of these mRNAs were unaltered in the spinal cord in the carrageenan model of hindpaw inflammation compared to control (Fig. 3a-d). Of the chemokines investigated in the PCX group, only *Cxcl6* mRNA was significantly higher than in the control group (*p* = 0.04, Mann-Whitney *U* test, FC = 2.6). Levels of *Cxcl6* mRNA in the carrageenan group were similar to the control group (Fig. 3e). There was a marked upregulation of genes encoding some chemokine receptors *Cxcr1* (*p* = 0.0082, FC = 9.7); *Cxcr5* (*p* = 0.0303, FC = 4.2); *Ccr1* (*p* = 0.0087, FC = 7.8); *Cx3cr1* (*p* = 0.0350, FC2.4,) in the PCX group compared to the control group (Fig. 3f-i), whilst in the carrageenan group these values were similar to the control. Consistent with the behavioural pain phenotype, expression of glutamate receptor *Grin2b* (NMDA receptor subunit) mRNA in the PCX group was higher than in the control group (*p* = 0.0140, Mann-Whitney *U* test, FC = 2.1; Fig. 3 j). The levels of glutamate receptor *Gria1* (AMPA receptor) and glutamate transporters Slc1a2 and Slc1a3 mRNAs in the PCX group were not different from control (data not shown). Similarly, the levels of *Grin2b*, *Gria1*, *Slc1a2* and *Slc1a3* mRNAs in carrageenan group were not different from the control group. Increases in mRNA expression of some excitatory molecules and pathways in the PCX group were counteracted by significant upregulation of mRNA for some anti-inflammatory molecules, *Socs2* (a suppressor of IL-6 signalling) (*p* = 0.0082, Mann-Whitney *U* test, FC = 4.6) and *Pparg* (an anti-inflammatory transcription factor peroxisome proliferator-activated receptor-γ) (*p* < 0.0043, Mann-Whitney *U* test, FC = 3.7) when comparing to the control group (Fig. 3k, l).

A comparison of changes in mRNA across PCX and carrageenan groups is reported in Supplemental file: Fig. 2. Although the levels of some mRNAs in the PCX model were lower than in the control group (e.g. *Hpgd*, *Casp1*, *Mmp9*, *Stat1*, *Pdcd4*, *Aitf1*, *Arg1*, *Gria1*, *Slc1a3* and *P2rx7*), statistical significance was not reached. With respect to markers of glia cell activation, there were trends towards a decrease in *Arg1* mRNA (maker for alternative-activated anti-inflammatory (M2) microglia), and an increase in mRNAs for glial cell markers in the PCX group (Supplemental file: Fig. 2).

The presence of any cause-effect relationship between the changes in the spinal gene expression and pain behaviour was investigated by undertaking correlation analyses. With the exception of *Cxcl6* and *Cxcr5*, the changes in mRNAs, which were significantly increased in the PCX group were negatively correlated with corresponding PWTs, indicating a positive association between increased expression in the dorsal horn of the spinal cord and the increased pain behaviour (Table 1).

**Table 1:** Correlations between behavioural pain responses and changes in mRNA expression

Cluster	Genes	Correlation of Δ^**CT**^ to mechanical pain threshold	
		Spearman *r*	*p* value
Pro-inflammatory cascades	*Tlr4*	−0.6398	0.0210
	*Nlrp1a*	−0.5966	0.0493
	*Tnfrsf1a*	−0.6621	0.0160
	*Tnfrsf1b*	−0.6989	0.0138
Anti-inflammatory cascades	*Soc2*	−0.6705	0.0143
	*Pparg*	−0.7541	0.0095
Central sensitisation	*Grin2b*	−0.6677	0.0149
Chemokine	*Cxcl6*	−0.5442	0.0704
Chemokine receptors	*Cxcr1*	−0.5671	0.0461
	*Cxcr5*	−0.4874	0.1021
	*Ccr1*	−0.7106	0.0170
	*CX3cr1*	−0.6174	0.0273
Resolvin synthetic enzyme	*Alox5ap*	−0.6989	0.0199
	*Cyp2j4*	−0.8145	0.0019
Resolvin degradation enzyme	*Ptgr1*	−0.6981	0.0151

Fold change of mRNA expression is Δ^CT^ of each gene of the PCX group relative to Δ^CT^ of the control group. The mechanical pain behaviour is expressed as the number of von Frey filaments changed (ΔvF) at day 28 compared to baseline. Group sizes, *n* = 11-13 rats

**Fig. 4:**
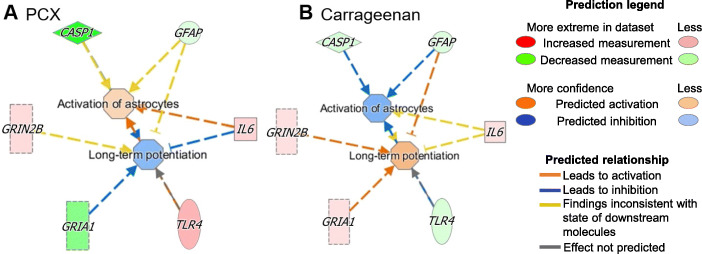
Bioinformatic analysis of spinal dorsal horn mRNA expression of PCX or carrageenan model. Predictions from ingenuity pathway analysis for the direction and intensity (variation in the colour shade) of the changes in genes involved in nervous system development and function based on the mRNA data for the dorsal horn of the spinal cord from PCX and carrageenan rats and their respective controls

To probe the significance of the global changes, rather than individual components, of these signalling pathways, we undertook ingenuity pathway analysis (IPA) core analysis of the mRNA expression data from the dorsal horn of the spinal cord collected using the microfluidic TaqMan array cards. The fold-change differences in gene expression in intervention groups (carrageenan model of acute pain and PCX model of CINP) relative to the control group were calculated, and a heat map was generated; Supplemental file: Fig. 3). IPA analysis revealed the most responsive cellular functions (of those selected for analysis) associated with our models of pain, versus control (Table 2), with the nervous system and development function having one of the highest levels of significance. Amongst the sub-functions that constitute the nervous system and development function, IPA predicted a downregulation in the activation of astrocytes in the carrageenan group and increased activation of astrocytes in the PCX group (Fig. 4a). IPA also predicted an upregulation of the long-term potentiation function in the carrageenan model (Fig. 4b), but a downregulation in the PCX model (Fig. 4a). These predictions are consistent with the known expansion of receptive field size of dorsal horn neurones in the carrageenan model of acute pain [40] and the decrease in the magnitude of evoked responses of WDR neurones in the PCX model demonstrated herein.

**Table 2:** The most affected cellular functions in the spinal dorsal horn of carrageenan and PCX models

	Carrageenan	PCX
Immune cell trafficking	7.74E−09	7.74E−09
Inflammatory response	7.74E−09	7.74E−09
Cellular compromise	1.56E−06	1.56E−06
Nervous system development and function	9.23E−06	9.23E−06
Hypersensitivity response	2.11E−05	2.11E−05
Neurological disease	2.46E−05	2.46E−05

Based on mRNA expression data quantified from the ipsilateral dorsal horn of rats received either carrageenan or paclitaxel compared to their respective controls (*n* = 5-7 in each group)

Data were generated using low-density microarray cards. Results shown represent the -log of *p* values calculated by Fisher’s exact test right tailed (*p* < 0.05)

### Resolution pathways and therapeutic potential of AT-RvD1 in the PCX model

**Fig. 5:**
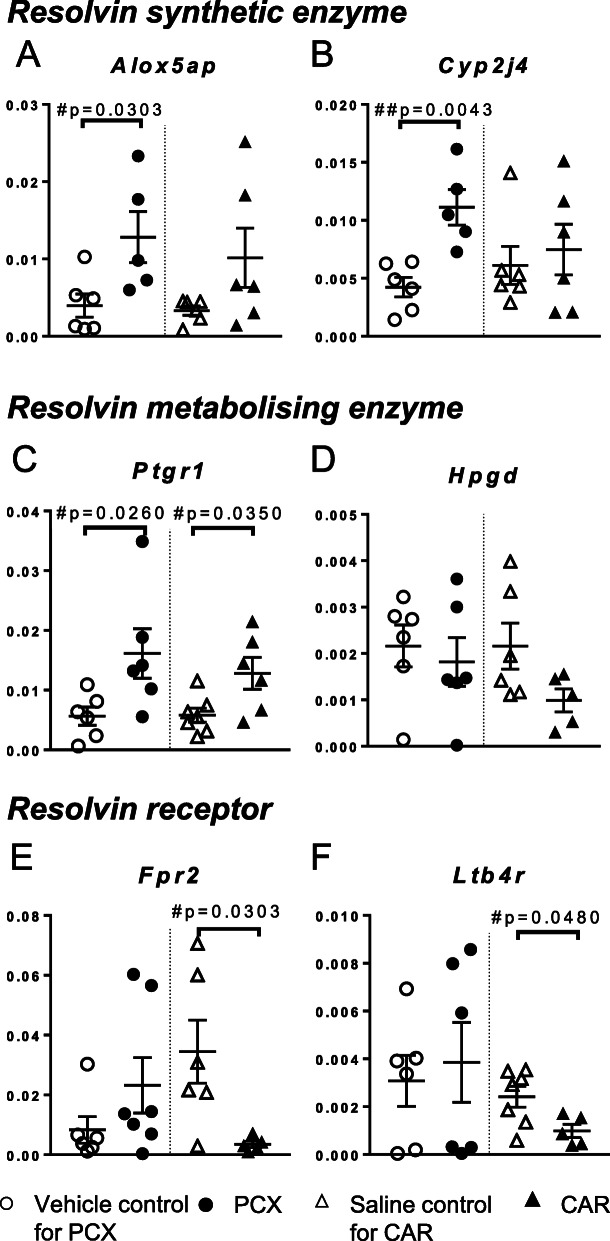
Spinal dorsal horn mRNA gene expression of in resolvin pathways in in PCX and carrageenan models. **a**, **b** Enzymes with known roles in the synthesis of resolvins. **c**, **d** Enzymes with known roles in the metabolism of the resolvins. **e**, **f** Two of the resolving receptors. Data expressed as median ± interquatile range of Δ^CT^ of PCX (●) and carrageenan (▲) compared to respective control (○/∆). Analysis with Mann-Whitney *U* test: #*p* < 0.05, ##*p* < 0.01 versus corresponding saline/vehicle, *n* = 5-7 in each group

Of the sixteen mRNAs encoding molecules and enzymes relevant to the resolution of inflammation, three were significantly upregulated in the PCX group compared to control: *Alox5ap* the lipoxygenase-5 (5LOX) activating protein (*p* = 0.0303, Mann-Whitney *U* test, FC = 3.2, Fig. 5a) and *Cyp2j4* (*p* = 0.0043, Mann-Whitney *U* test, FC = 3.4, Fig. 5b), which are enzymes essential in D- and E-series resolvin synthesis, and *Ptgr1* (prostaglandin reductase 1), which metabolises E-series resolvins (*p* = 0.0260, Mann-Whitney *U* test, FC = 2.8, Fig. 5c). Other enzymes involved in the synthesis (*Alox15*, *Alox5*, *Lta4h*) and degradation (*Hpgd*, *Cyp2e1* and *Cyp4f4*) of the resolvins were not statistically altered. There were trends towards an increase in the expression of genes encoding the resolvin receptors *Fpr2* and *Ltb4r* in the PXC model (Fig. 5e, f). However, in the carrageenan model of acute hindpaw inflammation, there were significant decreases in *Fpr2* and *Ltb4r* mRNA (Fig. 5e, f).

We attempted to unravel any cause-effect relationship between changes in the spinal expression of mRNAs encoding enzymes that contribute to the generation and catabolism of the resolvin molecules and pain behaviour by undertaking correlation analyses. Increased mRNA levels of *Alox5ap*, *Cyp2j4*, and *Ptgr1* were significantly correlated with increased pain behaviour in the PCX model (Supplemental file: Fig. 4), supporting a role in the resolution pathways in the PCX model. *Casp1* (encodes caspase-1 which cleaves ProIL-1β to active IL-1β) was identified by the IPA analysis and was significantly correlated with *Alox5*, *Alox5ap* and *Ptgr1* (Supplemental file: Table 4). In addition, the chemokine receptor *Ccr7* was correlated with *Alox5ap* and *Ptgr1* and the RvE1 receptor *Ltb4r* (Supplemental file: Table 4).

### Selective effects of spinal AT-RvD1 in the PCX model of CINP

**Fig. 6:**
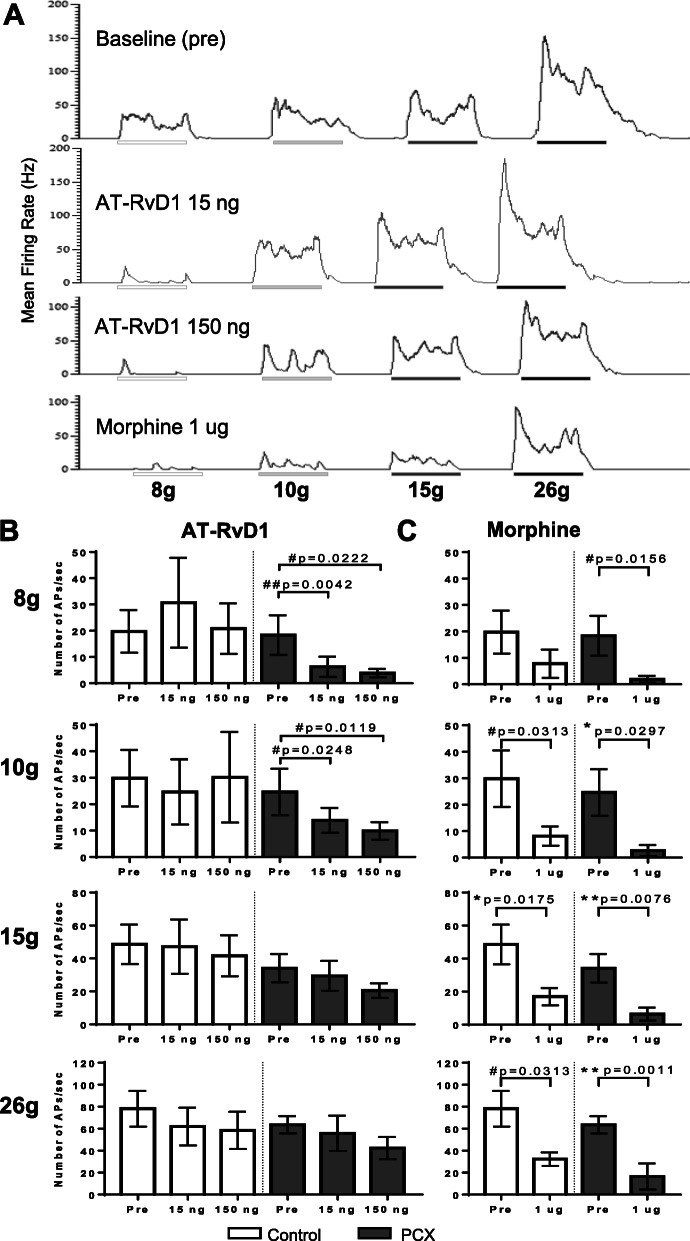
Effects of spinal AT-RvD1 versus morphine on mechanically evoked WDR neuronal responses of PCX and control rats. **a** Representative electrophysiological traces of mechanical (8, 10, 15 and 26 g) evoked responses of a WDR neurone of a PCX-treated rat at baseline and following the spinal application of AT-RvD1 15 ng, 150 ng and morphine 1 μg. **b** In PCX rats, AT-RvD1 (15 and 150 ng) dose-relatedly inhibited 8 g and 10 g, but not 15 g and 26 g, evoked responses of WDR neurones, compared to pre-drug responses (pre). AT-RvD1 had no significant effect on mechanically evoked responses of WDR neurones in control rats. Analysis with repeated measure ANOVA with Dunnett’s post hoc test for AT-RvD1 15 and 150 ng versus pre-drug responses: *p < 0.05, **p < 0.01, ***p < 0.001 (*n* = 6-9/group). **c** Morphine suppressed low and high weight mechanical evoked responses of WDR neurones in both control and PCX rats. Analysis with either a paired *t* test (*) or Wilcoxon test (#) for morphine versus pre-drug responses: * or #*p* < 0.05,** or ##*p* < 0.01, (*n* = 6-7 neurones in each group). APs, action potentials. Most data expressed as median ± interquatile range except 15 g responses in control group; 10 g, 15 g, and 26 g responses in PCX morphine groups which data were normally distributed

The significant changes in mRNA levels encoding key enzymes involved in the synthesis and catabolism of the resolvins in the dorsal horn of the spinal cord lead us to investigate whether upregulation of this pathway had functional effects in the PCX model. Thus, we tested whether augmentation of the resolvin pathway, by the delivery of a potent ligand, suppressed the spinal nociceptive signalling in this model. Representative electrophysiological traces of mechanically evoked responses of a WDR neurone in a PCX-treated rat at baseline and following the spinal application of AT-RvD1 (15 and 150 ng) and morphine (1 μg) are shown in Fig. 6a, c. AT-RvD1 (15 and 150 ng) dose-relatedly inhibited low intensity (8 g and 10 g) evoked responses of WDR neurones (8 g, *p* = 0.0012; Friedman statistic 11.9; 10 g, *p* = 0.008, Friedman statistic 9.25) in PCX, but not in the control group (Fig. 6b). The magnitude of the effects of the two doses of AT-RvD1 on 8 g evoked responses in PCX rats were comparable to 1 μg of morphine (73 ± 5% and 73 ± 7% versus 84 ± 4% inhibition, *p* > 0.05, Wilcoxon test; Fig. 6b, c). Inhibitory effects of the spinal AT-RvD1 (15 and 150 ng) on 10 g mechanically evoked responses in the PCX group were smaller (35 ± 18 and 46 ± 11% inhibition, respectively), about half the effect of 1 μg of morphine (84 ± 4% inhibition, Fig. 6b, c). The peak inhibitory effects of AT-RvD1 on mechanically evoked responses were in the range of 15-30 min post application. Unlike spinal morphine, spinal AT-RvD1 did not significantly alter the 15 and 26 g evoked responses of WDR neurones in PCX rats (Fig. 6b, c).

## Discussion

The PCX model of CINP was associated with marked changes in the neurophysiological responses of WDR neurones and increases in the mRNA expression of Grin2b and cytokine and chemokine molecules that drive pro-inflammatory signalling in the dorsal horn of the spinal cord. Ingenuity pathway analysis predicted increased activation of astrocytes and decreased long-term potentiation in the dorsal horn in the PCX model. Significant changes in mRNA expression of enzymatic pathways involved in the synthesis and catabolism of the resolvins were evident in the dorsal horn in the PCX model. Spinal administration of the resolvin AT-RvD1 inhibited low weight mechanical-evoked responses of WDR neurones in PCX rats, but not in controls. Collectively, these data support the therapeutic targeting of the resolution pathways as an intervention aimed at ameliorating aberrant neuropathic pain responses associated with chemotherapeutic treatments.

The PCX model of CINP was associated with changes in physiological responses, including lowered thresholds for electrical Aβ and C-fibre responses of WDR neurones. Following natural hindpaw stimulation, a significantly higher proportion of WDR neurones exhibited spontaneous activity and polymodality (responsivity to acetone and post-discharge firing following low weight 8 g-mechanical stimulation) in the PCX model. Our findings are consistent with the report of spontaneous ectopic activity of DRG somata in models of CINP [41] and DRGs taken from people with neuropathic pain [42]. Despite changes to the normally non-painful stimuli, the magnitudes of noxious mechanical evoked responses of WDR neurones were (generally) reduced in the PCX model, consistent with other models of neuropathic pain [30, 43]. Types of anaesthesia are known to influence WDR neurones neurophysiological properties [44], and may account for some differences seen between these studies and previous work using urethane [12]. Spinal plasticity leading to altered responses to normally non-painful mechanical stimuli in the PCX model may be underpinned by our reported changes in glutamate receptor signalling, specifically increased Grin2b mRNA, which encodes the glutamate ionotropic NMDA receptor subunit GluN2B, in the dorsal horn of the spinal cord in the PCX model. Our data provide new evidence for changes in both the afferent input and synaptic circuitry, which may underpin changes in the response properties of the dorsal horn neurones and the increased polymodality in the PCX model.

Gene card analysis and ingenuity pathway analysis identified marked changes in inflammatory signalling pathways in the PCX model, although we acknowledge that not all changes in mRNA levels may lead to altered protein levels, this analysis provides novel insight into the underlying pathway changes in this model. IPA analysis revealed that the nervous system and development function was one of the cellular functions with the highest level of significance for the PCX model, compared to the control group. Based on the integration of overall changes in all genes (e.g. *Casp1*, *Gria1*, *Tlr4*) that contribute to the sub-function of astrocyte activation, IPA predicted increased activation of astrocytes in the dorsal horn of the spinal cord in the PCX group compared to the control group, and a downregulation in the activation of astrocytes in the dorsal horn of the spinal cord in the carrageenan group compared to the control. These predictions are consistent with the evidence for the role of astrocytes in modulating the spinal processing of sensory inputs in models of chronic pain (see the “Introduction” section).

The significant increase in the expression of *Nlrp1a* mRNA in the dorsal horn suggests a possible involvement of the inflammasome in the molecular responses of the PCX model. Although the role of the NLRP1 inflammasome in neuropathic pain has previously been reported [45], here we extend this to a model of CINP. Activation of inflammasomes results in the cleavage of caspase-1, which initiates several down-stream pro-inflammatory pathways, including IL-1β signalling. IPA also predicted decreases in caspase-1 activity, consistent with activation of inflammasomes. In line with our reported trend towards increased expression of *Nlrp3* in the PCX model, previous studies have assigned a role for spinal *Nlrp3* in models of neuropathic pain [46]. Alongside the identification of new potential mechanisms, our study confirmed changes in mRNA expression of chemokines and receptors at this level (Supplemental file: Figs. 2 and 3). There was a significant upregulation of *Cx3cr1* receptor mRNA, but not its ligand *Cx3cl1* (fractalkine) mRNA, in the PCX model, consistent with data in the CCI model of neuropathic pain [47]. A significant upregulation of mRNA for *Cxcl6* and the corresponding receptor *Cxcr1* in the PCX model, consistent with known roles in sensitisation of pain pathways at central sites [48] was also evident. Gene expression of *Tlr4* and TNF-α receptors, *Tnfrsf1a* and *Tnfrsf1b* were significantly increased in the dorsal horn of the spinal cord in the PCX model, compared to controls, consistent with their known role in spinal nociceptive hyperexcitability [49] and pain behaviour in this model [7]. Despite mRNAs for *Socs2* and *Pparg*, which are anti-inflammatory, being significantly upregulated in the spinal cord, mechanical sensitivity was still evident in the PCX model. These data suggest that endogenous mechanisms to counter spinal neuroinflammation may be enhanced in PCX-induced neuropathic pain, but may not be sufficient to surmount the pro-inflammatory mechanisms leading to central sensitisation and the manifestation of aberrant pain behaviour.

To further advance our knowledge of the impact of CINP on spinal nociceptive processing and potential novel targets for treatment, we investigated whether genes with known roles in the generation/catabolism of the resolvins were altered in the PCX model. Levels of genes encoding several enzymes that sequentially catalyse the generation of D- and E-series resolvins from their precursors (COX-2, 15-LOX, 5-LOX, FLAP, LTA4H and CYP2J4) are shown in Supplemental file: Fig. 5 [50, 51]. In vitro studies suggest that RvD1 generation is dependent on FLAP activity [50]. In our study, the levels of *Alox5ap* (which encodes FLAP) mRNA were significantly elevated in the PCX model, which is predicted to result in increased levels of RvD1. However, several genes that control enzymatic inactivation of the resolvins were also significantly upregulated in the PCX model. Leukotriene B4 12-hydroxydehydrogenase (LTB4DH, encoded by *Ptgr1*) metabolises E-series resolvins [51] and mRNA levels were significantly increased in the PCX group. However, mRNA levels of hydroxyprostaglandin dehydrogenase (HPGD, encoded by *Hpgd*), which metabolises both D- and E-series resolvins [52, 53] were not altered in the PCX model. CYPs are a large family of enzymes metabolising xenobiotics and endogenous lipids [54] whose isoforms are expressed in pain pathways [55, 56]. Of the CYP genes studied, two have been implicated in resolvin synthesis (CYP2J4 and CYP2C7) and two in resolvin degradation (CYP4F4 and CYP2E1) [52, 53]. The increased expression of spinal *Cyp2j4* in the PCX model reported herein also predicts increased resolvin synthesis, and is consistent with the increased expression of a mouse homologue of rat *Cyp2j4* in DRG from PCX mice [56]. These enzymes, however, are involved in the generation of a broad spectrum of bioactive lipids. For example, *Cyp2j4* produces 9,10-epoxy-12Z-octadecenoic acid, which sensitises TRPV1 in DRG culture and reduces thermal and mechanical thresholds in vivo [56]. We do recognise that measurements of the resolvins are required to confirm that changes in gene expression have metabolic consequences. However, these molecules are relatively unstable, and coupled with the low levels of resolvins reported [18, 57] their detection in discrete regions such as the ipsilateral dorsal horn of the spinal cord may be challenging [58].

Despite the changes in the expression of the genes encoding enzymatic routes involved in resolvins metabolism, mRNA levels of some key resolvin receptors were unaltered in the dorsal horn in the PCX model, which lends support for an intervention that targets the resolution pathways in the PCX model of CINP. On the basis of the changes in gene expression, we considered that the effects of AT-RvD1, which is metabolised predominantly by HPGD [53] but not LTB4DH, worthy investigating. The targets for AT-RvD1 are FPR2/ALX and GPR32 receptors [19], FPR2/ALX receptor is expressed in rodents and thought to mediate the biological effects of AT-RvD1 [59]. Previously, we demonstrated that the inhibitory effects of AT-RvD1 are blocked by the FPR2/ALX receptor antagonist butoxycarbonyl-Phe-Leu-Phe-Leu-Phe (BOC-2) suggesting FPR2/ALX receptor-dependent inhibition [22]. In the present study, mRNA expression of FPR2/ALX receptor was unaltered in the dorsal horn of the spinal cord in the PCX model and spinal administration of AT-RvD1 inhibited low-weight mechanically evoked responses of spinal neurones, with a magnitude of effect comparable to that of spinal morphine. In contrast to the PCX model, FPR2/ALX mRNA was downregulated in the dorsal horn of the spinal cord from the acute carrageenan model of inflammation, which may reflect high concentrations of SPMs produced during the late phase of inflammation [60].

Unlike morphine, AT-RvD1 did not alter low-weight mechanically evoked responses of WDR neurones in the control group and did not alter the high-intensity mechanically evoked responses of neurones in either PCX or control groups. The marked inhibitory effects of AT-RvD1 on low mechanical evoked responses of WDR neurones, which are relayed by A-fibres, suggest that targeting the spinal resolution pathways will ameliorate pain responses evoked by these types of stimuli, which are particularly problematic for people suffering from CINP [3]. On the basis of the study by Luo et al. (21) reporting comparable effects of intrathecal RvD1 and RvD2, but not RvD5, in male and female mice in the PCX model, we only undertook our study using AT-RvD1 in male rats. However, we acknowledge this wider implications of our study and is a limitation of our work. The lack of effect of AT-RvD1 on WDR neuronal responses to higher weight mechanical stimuli suggests that treatments acting through the resolvin pathways are unlikely to modulate normal physiological high-intensity nociceptive processing, ensuring that this major protective pathway remains intact and functional. Indeed, our previous study reported no effect of AT-RvD1 on electrically evoked C-fibre responses of WDR neurones in naïve rats [22] and behavioural studies reported no change in baseline threshold responses following spinal RvD1 treatment [61]. Our data build upon the recent report that intrathecal injection of RvD1 attenuates mechanical hypersensitivity in PCX-treated mice [21], and provides neurophysiological mechanisms underlying these antihyperalgesic effects seen in models of CINP.

## Conclusion

The treatment of CINP remains a clinical challenge, as existing treatments either have poor side-effect profiles, limited efficacy or in the case of opioid ligands exhibit tolerance and potential addiction. The magnitude of the inhibitory effects of AT-RvD1 on low weight mechanically evoked responses of WDR neurones was comparable to those of spinal morphine. Unlike opioid receptor agonists, there is no evidence for the development of tolerance to the effects of systemic resolvin treatment in a model of chronic pain [58]. In conclusion, our study supports the view that targeting the spinal resolvin pathway has the therapeutic potential to thwart aberrant spinal neuronal responses to low weight mechanical stimuli, which are known to uniquely activate pain circuits in patients living with chemotherapy-induced neuropathic pain.

## Supplementary information

Additional file 1: **Table S1.** Rat group sizes of studies. **Table S2.** Selected target and reference genes for TLDA study. **Table S3.** Evoked responses of WDR neurones in PCX versus control rats. **Table S4.** Correlations between genes involved in the resolvin system and other selected genes studied. **Figure S1.** Behavioural pain responses in the PCX model and carrageenan model. **Figure S2.** mRNA expression profile in rat ipsilateral dorsal horn of the spinal cord in the PCX and carrageenan models. **Figure S3.** Heat map of individual mRNA abundance relative to appropriate control (saline) in the ipsilateral dorsal horn of the spinal cord of rats following induction of the inflammatory pain model (carrageenan, n=6) and the model of chemotherapy induced neuropathic pain (PCX n= 7). Red signifies greater relative abundance, while green signifies less relative abundance. **Figure S4.**. Correlations between pain behavior (number of von Frey filament changed from baseline (ΔvF)) and expression levels of genes involving generation and catabolism of the resolvin molecules. **Figure S5.** The synthetic and catabolic pathways for the resolvins. (DOCX 4227 kb)

## Data Availability

The datasets used and/or analysed during the current study are available from the corresponding author on reasonable request.

## Abbreviations

5LOX: Ipoxygenase-5
8gPD: Generation of post-discharge after low-intensity mechanical stimulation by 8 von Frey hair
Aitf1: Allograft inflammatory factor 1; ionised calcium-binding adaptor molecule 1 or allograft inflammatory factor 1 (IbA1)
Alox15: Arachidonate 15-lipoxygenase activating protein
Alox5: Arachidonate 5-lipoxygenase activating protein
Alox5ap: Arachidonate 5-lipoxygenase activating protein; FLAP (5-lipoxygenase activating protein )
AMPA: α-amino-3-hydroxy-5-methyl-4-isoxazolepropionic acid
AR: Acetone responsiveness
Arg1: Arginase 1
AT-RvD1: Aspirin-triggered resolvin D1
Casp1: Caspase 1
CCR1: Chemokine (C-C motif) receptor 1
CINP: Chemotherapy-induced neuropathic pain
Ct: Cycle threshold
CX3CR1: Chemokine (C-X3-C motif) receptor 1
CXCL6: Chemokine (C-X-C motif) ligand 6
CXCR1: Chemokine (C-X-C motif) receptor 1
CXCR5: Chemokine (C-X-C motif) receptor 5
CYP2E1: Cytochrome P450, family 2, subfamily e, polypeptide 1
CYP2J4: Cytochrome P450, family 2, subfamily j, polypeptide 4
CYP4F4: Cytochrome P450, family 4, subfamily f, polypeptide 4
DRG: Dorsal root ganglion
ΔvF: The number of von Frey filament changed from baseline
FC: Fold change
Fpr2: Formyl peptide receptor 2 (FPR2/ALX or ALX receptor)
GABA: Gamma-aminobutyric acid
Gria1: Glutamate receptor, ionotropic, AMPA 1, glutamate ionotropic receptor AMPA type subunit 1 (GluR1)
Grin2b: Glutamate receptor, ionotropic, N-methyl D-aspartate 2B; glutamate ionotropic receptor NMDA type subunit (GluN2B)
Hpgd: Hydroxyprostaglandin dehydrogenase
Lta4h: Leukotriene A4 hydrolase
Ltb4r: Leukotriene B4 receptor or BLT-1 receptor
MMP1: Matrix metallopeptidase 9
NLRP1A: NOD-like receptor family, pyrin domain-containing 1A
NMDA: N-methyl-D-aspartate
P2rx7: Purinergic receptor P2X, ligand-gated ion channel, 7 (P2X7)
PCX: Paclitaxel
PD: Post-discharge
Pdcd4: Programmed cell death 4
PPARγ: Peroxisome proliferator-activated receptor-γ
Ptgr1: Prostaglandin reductase 1
PWT: Paw withdrawal threshold
RvD1: Resolvin D1
RvE1: Resolvin E1
SA: Spontaneous activity
Slc1a2: Solute carrier family 1 (glial high-affinity glutamate transporter), member 2; glutamate transporter1 (GLT1)
Slc1a3: Solute carrier family 1 (glial high affinity glutamate transporter), member 3; glutamate-aspatate transporter (GLAST)
SOCS2: Suppressor of cytokine signaling 2
SPMs: Secialised pro-resolving mediators
Stat1: Signal transducer and activator of transcription 1
TLDA: TaqMan® Low-Density Array
TLR: Toll-liked receptor
TNF-α: Tumour necrosis factor-α
TNFRSF1A: Tumour necrosis factor receptor superfamily, member 1A
TNFRSF1B: Tumour necrosis factor receptor superfamily, member 1B
TRPV1: Transient receptor potential cation channel subfamily V member 1
WDR: Wide dynamic range
WU: Wind-up
