# Supplementary material for: Spinal neuronal excitability and neuroinflammation in a model of chemotherapeutic neuropathic pain: targeting the resolution pathways
Source: J Neuroinflammation. 2020 Oct 23;17:316. doi: 10.1186/s12974-020-01997-w (PMC7585293; doi:10.1186/s12974-020-01997-w)
Supplement: Supplementary file 1 — Table S1. Rat group sizes of studies. Table S2. Selected target and reference genes for TLDA study. Table S3. Evoked responses of WDR neurones in PCX versus control rats. Table S4. Correlations between genes involved in the resolvin system and other selected genes studied. Figure S1. Behavioural pain responses in the PCX model and carrageenan model. Figure S2. mRNA expression profile in rat ipsilateral dorsal horn of the spinal cord in the PCX and carrageenan models. Figure S3. Heat map of individual mRNA abundance relative to appropriate control (saline) in the ipsilateral dorsal horn of the spinal cord of rats following induction of the inflammatory pain model (carrageenan, n=6) and the model of chemotherapy induced neuropathic pain (PCX n= 7). Red signifies greater relative abundance, while green signifies less relative abundance. Figure S4.. Correlations between pain behavior (number of von Frey filament changed from baseline (ΔvF)) and expression levels of genes involving generation and catabolism of the resolvin molecules. Figure S5. The synthetic and catabolic pathways for the resolvins. (DOCX 4227 kb) [file 12974_2020_1997_MOESM1_ESM.docx]

**Table S1 Rat group sizes of studies**

| Method | Model | Spinal treatment | Group Size |
| --- | --- | --- | --- |
| Electrophysiology | Intraperitoneal vehicle | AT-RvD1-Morphine | 16 |
|  | Intraperitoneal PCX | AT-RvD1-Morphine | 15 |
| Gene expression using TLDA | Intraplantar saline | NA | 6 |
|  | Intraplantar carrageenan | NA | 7 |
|  | Intraperitoneal vehicle | NA | 7 |
|  | Intraperitoneal PCX | NA | 7 |
|  |  | Total | 58 |

NA=not applicable

**Table S2** Selected target and reference genes for TLDA study

| ***Gene name*** | ***Gene alias*** | **Protein name** | **ABI assay ID** |
| --- | --- | --- | --- |
| ***Resolvin System*** | | | |
| *Alox15* | *12-LOX;15-LOX;Alox12;Alox12l* | arachidonate 15-lipoxygenase (15-LOX); 15-LOX (15-lipoxygenase or 12/15-lipoxygenase) | Rn00578743_m1 |
| *Alox5* | *LOX5A* | arachidonate 5-lipoxygenase; 5-LOX (5-lipoxygenase) | Rn00563172_m1 |
| *Alox5ap* | *-* | arachidonate 5-lipoxygenase activating protein; FLAP (5-lipoxygenase activating protein ) | Rn00568506_m1 |
| *Cmklr1* | *Chemr23* | chemokine-like receptor 1 or ChemR23 | Rn00573616_s1 |
| *Cyp2c7* | *Cyp2c39* | cytochrome P450, family 2, subfamily c, polypeptide 7;Cytochrome P450, family 2, subfamily c, polypeptide 7-like | Rn01529602_mH |
| *Cyp2e1* | *Cyp2e* | cytochrome P450, family 2, subfamily e, polypeptide 1 (CYP2E1) | Rn00580624_m1 |
| *Cyp2j4* | *CYP2J2* | cytochrome P450, family 2, subfamily j, polypeptide 4; also known as CYP2J4 | Rn00576482_m1 |
| *Cyp4f4* | *-* | cytochrome P450, family 4, subfamily f, polypeptide 4 (CYP4F4) | Rn01408362_g1 |
| *Fpr2* | *Fpr-rs2;Fprl1* | formyl peptide receptor 2 (FPR2/ALX or ALX receptor) | Rn03037051_gH |
| *Gpr18* | *-* | G protein-coupled receptor 18 (Gpr18) | Rn01493247_m1 |
| *Hpgd* | *-* | hydroxyprostaglandin dehydrogenase 15 (NAD) (HPGD); 15-PGDH (15-hydroxyprostaglandin dehydrogenase) | Rn00577775_m1 |
| *Lta4h* | *-* | leukotriene A4 hydrolase (LTA4H) | Rn01503878_m1 |
| *Ltb4r* | *Ltb4r1* | leukotriene B4 receptor or BLT-1 | Rn00572209_s1 |
| *Ptges* | *Pges* | prostaglandin E synthase; microsomal prostaglandin E synthase-1 (mPGS-1) | Rn00572047_m1 |
| *Ptgr1* | *Dig1;Ltb4dh* | prostaglandin reductase 1 (PTGR1); leukotriene B4 12-hydroxydehydrogenase (LTB4DH) | Rn00593950_m1 |
| *Ptgs2* | *COX-2;Cox2* | prostaglandin-endoperoxide synthase 2; cycloocxygenase-2 (COX-2) | Rn00568225_m1 |
| **Proinflammatory cascades** | | | |
| *Casp1* | *Ice;Il1bc* | caspase 1 | Rn00562724_m1 |
| *Cd14* | *-* | Monocyte differentiation antigen CD14 | Rn00572656_g1 |
| *Cd40lg* | *Cd40l;Tnfsf5* | CD40 ligand (CD40L) | Rn00584362_m1 |
| *Chuk* | *Ikbka;Ikka* | conserved helix-loop-helix ubiquitous kinase; inhibitor of nuclear factor kappa-B kinase alpha (IKKα) | Rn01444501_m1 |
| *Il17a* | *CTLA-8;IL-17;IL-17A;Il17* | interleukin 17A (IL-17A) | Rn01757168_m1 |
| *Il1b* | *-* | interleukin 1 beta ( IL-1β) | Rn00580432_m1 |
| *Il1r1* | *-* | interleukin 1 receptor, type I (IL-1β receptor) | Rn00565482_m1 |
| *Il6* | *ILg6;Ifnb2* | interleukin 6 (IL-6) | Rn99999011_m1 |
| *Il6r* | *IL6R1;Il6ra* | interleukin 6 receptor, ligand binding subunit | Rn01495381_m1 |
| *Il8* | *Nap1, Cxcl8* | similar to Nef associated protein 1; IL-8 or C-X-C motif chemokine ligand 8 (CXCL8) | Rn01437056_m1 |
| *Irak1* | *RGD1563841* | interleukin-1 receptor-associated kinase 1 (IRAK) Checked mRNA changeable(Li et al., 2011) | Rn01193545_m1 |
| *Jak2* | *-* | Janus kinase 2 (Jak2) | Rn00676341_m1 |
| *Mmp2* | *-* | matrix metallopeptidase 2 | Rn01538170_m1 |
| *Mmp9* | *-* | matrix metallopeptidase 9 | Rn00579162_m1 |
| *Myd88* | *-* | myeloid differentiation primary response 88 (MyD88) | Rn01640049_m1 |
| *Nfkb1* | *EBP-1;NF-kB* | nuclear factor of kappa light polypeptide gene enhancer in B-cells 1; nuclear factor kappa B subunit 1 ( NF-κB subunit 1); p50 | Rn01399583_m1 |
| *Nfkbia* | *RL/IF-1* | nuclear factor of kappa light polypeptide gene enhancer in B-cells inhibitor, alpha; NF-κB inhibitor alpha (IκBα) | Rn01473657_g1 |
| *Nlrp1a* | *Nalp1;Nlrp1* | NOD-like receptor family, pyrin domain containing 1A (NLRP1) | Rn01467482_m1 |
| *Nlrp3* | *Cias1* | NOD-like receptor family, pyrin domain containing 3 (NLRP3) | Rn04244620_m1 |
| *Otulin* | *Fam105b* | OTU deubiquitinase with linear linkage specificity or ubiquitin thioesterase otulin | Rn00573991_m1 |
| *Pdcd4* | *Dug* | programmed cell death 4 | Rn00573954_m1 |
| *Rela* | *NFkB* | v-rel avian reticuloendotheliosis viral oncogene homolog A; RELA proto-oncogene, NF-κB subunit p65 | Rn01502266_m1 |
| *Socs1* | *Cish1;Socs-1* | suppressor of cytokine signaling 1 (SOCS-1) | Rn00595838_s1 |
| *Socs2* | *Cish2;Socs-2* | suppressor of cytokine signaling 2 (SOCS-2) | Rn00589521_m1 |
| *Socs3* | *Cish3;Socs-3;Ssi-3* | suppressor of cytokine signaling 3 (SOCS-3) | Rn00585674_s1 |
| *Stat1* | *DD6G4-4* | signal transducer and activator of transcription 1 (STAT-1) | Rn00583505_m1 |
| *Stat3* | *-* | signal transducer and activator of transcription 3 (STAT-3); acute-phase response factor) | Rn00680715_m1 |
| *Timp1* | *TIMP-1;Timp* | TIMP metallopeptidase inhibitor 1 | Rn01430873_g1 |
| *Timp2* | *-* | TIMP metallopeptidase inhibitor 2 | Rn00573232_m1 |
| *Tlr4* | *-* | toll-like receptor 4 (TLR4) | Rn00569848_m1 |
| *Tnf* | *RATTNF;TNF-alpha;Tnfa* | tumor necrosis factor;tumor necrosis factor-alpha ( TNF-α) | Rn00562055_m1 |
| *Tnfrsf1a* | *TNFR-1;Tnfr1* | tumor necrosis factor receptor superfamily, member 1a (TNFR1) | Rn01492348_m1 |
| *Tnfrsf1b* | *Tnfr2* | tumor necrosis factor receptor superfamily, member 1b (TNFR2) | Rn00709830_m1 |
| *Traf3* | *-* | TNF receptor-associated factor 3 (TRAF3) | Rn01429852_m1 |
| *Traf6* | *-* | TNF receptor-associated factor 6 (TRAF6); E3 ubiquitin protein ligase | Rn01512911_m1 |
| **Anti-inflammatory cascades** | | | |
| *Aif1* | *BART-1;Bart1;iba1;mrf-1* | allograft inflammatory factor 1; ionised calcium-binding adaptor molecule 1 or allograft inflammatory factor 1 (IbA1) | Rn00574125_g1 |
| *Arg1* | *-* | arginase 1 (Arg1) | Rn00691090_m1 |
| *Cd163* | *-* | CD163 molecule | Rn01492519_m1 |
| *Chi3l3* | *Chi3l4;Chil3;rYM1olf* | chitinase 3-like 3 (Chi3l3) or Ym1 | Rn01523660_g1 |
| *Gata3* | *-* | GATA binding protein 3 *(GATA3)* | Rn00484683_m1 |
| *Gfap* | *-* | glial fibrillary acidic protein (GFAP) | Rn01253033_m1 |
| *Il10* | *IL10X* | interleukin 10 (IL-10) | Rn00563409_m1 |
| *Il13* | *-* | interleukin 13 (IL-13) | Rn00587615_m1 |
| *Il4* | *Il4e12* | interleukin 4 (IL-4) | Rn99999010_m1 |
| *Itgam* | *Cd11b* | integrin subunit alpha M | Rn00709342_m1 |
| *Kcnip3* | *Csen;Dream;rKChIP3* | Kv channel interacting protein 3; calsenilin; downstream regulatory element-antagonist modulator (DREAM) | Rn00583484_m1 |
| *Ppara* | *PPAR* | peroxisome proliferator activated receptor alpha (PPAR-α) | Rn00566193_m1 |
| *Ppard* | *Pparb* | peroxisome proliferator-activated receptor delta (PPAR-δ) | Rn00565707_m1 |
| *Pparg* | *-* | peroxisome proliferator-activated receptor gamma (PPAR-γ) | Rn00440945_m1 |
| *Retnla* | *Himf* | resistin like alpha (*Retnla*) or Fizz1 | Rn00584229_m1 |
| *S100b* | *S100P* | S100 calcium binding protein B | Rn04219408_m1 |
| *Tgfb1* | *Tgfb* | transforming growth factor, beta 1 (TGF-β1) | Rn00572010_m1 |
| *Trem2* | *-* | triggering receptor expressed on myeloid cells 2 (Trem2) | Rn01512170_m1 |
| **Markers for central sensitisation** | | | |
| *Gria1* | *GluA1;gluR-A* | glutamate receptor, ionotropic, AMPA 1, glutamate ionotropic receptor AMPA type subunit 1 (GluR1) | Rn00709588_m1 |
| *Grin2b* | *GluN2B* | glutamate receptor, ionotropic, N-methyl D-aspartate 2B; glutamate ionotropic receptor NMDA type subunit (GluN2B) | Rn00680474_m1 |
| *Nos2* | *Nos2a;iNos* | nitric oxide synthase 2; inducible nitric oxide synthase (iNOS) | Rn00561646_m1 |
| *P2rx7* | *-* | purinergic receptor P2X, ligand-gated ion channel, 7 (P2X7) | Rn00570451_m1 |
| *Slc1a2* | *Eaat2;Glt;Glt-1* | solute carrier family 1 (glial high affinity glutamate transporter), member 2; glutamate transporter1 (GLT1) | Rn00691548_m1 |
| *Slc1a3* | *EAAT1;GLAST;GLAST-1;GluT-1* | solute carrier family 1 (glial high affinity glutamate transporter), member 3; glutamate-aspatate transporter (GLAST) | Rn01402419_g1 |
| **Chemokines and chemokine receptors** | | | |
| *Ccl2* | *MCP-1;Scya2;Sigje* | chemokine (C-C motif) ligand 2 | Rn00580555_m1 |
| *Ccl21* | *Ccl21b* | chemokine (C-C motif) ligand 21 | Rn01764651_g1 |
| *Ccl7* | *-* | chemokine (C-C motif) ligand 7 | Rn01467286_m1 |
| *Ccr1* | *-* | chemokine (C-C motif) receptor 1 | Rn00571950_s1 |
| *Ccr2* | *-* | chemokine (C-C motif) receptor 2 | Rn01637698_s1 |
| *Ccr3* | *Cmkbr3* | chemokine (C-C motif) receptor 3 | Rn02134292_s1 |
| *Ccr7* | *-* | chemokine (C-C motif) receptor 7 | Rn02758813_s1 |
| *Ctss* | *-* | cathepsin S *Checked mRNA changeable* | Rn00569036_m1 |
| *Cx3cl1* | *Cx3c;Scyd1* | chemokine (C-X3-C motif) ligand 1 | Rn00593186_m1 |
| *Cx3cr1* | *Rbs11* | chemokine (C-X3-C motif) receptor 1 | Rn02134446_s1 |
| *Cxcl1* | *CINC-1;Gro1* | chemokine (C-X-C motif) ligand 1 | Rn00578225_m1 |
| *Cxcl13* | *-* | chemokine (C-X-C motif) ligand 13 | Rn01450028_m1 |
| *Cxcl6* | *Cxcl5* | chemokine (C-X-C motif) ligand 6 | Rn00573587_g1 |
| *Cxcr1* | *Il8ra* | chemokine (C-X-C motif) receptor 1 | Rn00570857_s1 |
| *Cxcr2* | *Cmkar2;Il8rb* | chemokine (C-X-C motif) receptor 2 | Rn02130551_s1 |
| *Cxcr5* | *Blr1;NLR* | chemokine (C-X-C motif) receptor 5 | Rn02132880_s1 |
| **Reference genes** | | | |
| *18s rRNA* | *18s rRNA* | - | Hs99999901_s1 |
| *Actb* | *Actx* | actin, beta (β-actin) | Rn00667869_m1 |
| *Gapdh* | *BARS-38;Gapd* | glyceraldehyde-3-phosphate dehydrogenase | Rn01749022_g1 |
| *Hprt1* | *Hgprtase;Hprt* | hypoxanthine phosphoribosyltransferase 1 | Rn01527840_m1 |
| *Mapk6* | *ERK3* | mitogen-activated protein kinase 6 | Rn00581152_m1 |

**Table S3 Evoked responses of WDR neurones in PCX versus control rats.**

Characteristics of spinal WDR neurones recorded on day 28-32 following intraperitoneal injection of PCX (2 mg/kg on four alternate days) or control treatment. Electrical-evoked responses of the PCX group compared to control. Mechanical-evoked responses of WDR neurones, responses to15 g von Frey stimulation of the hindpaw were significantly lower in the PCX group compared to control.

|  | **Control** | | |  | **PCX** | | | **PCX**  **(% of control)** |
| --- | --- | --- | --- | --- | --- | --- | --- | --- |
| Number of analysed neurones | 24 | | |  | 27 | | | NA |
| Electrical-evoked  responses (number of APs) |  | | |  |  | | |  |
| - Aβ^a^ | **146** | **±** | **7** |  | **142** | **±** | **4** | **97** |
| - Aβ^b^ | 149 | ± | 8 |  | 153 | ± | 8 | 103 |
| - Aδ^b^ | 165 | ± | 12 |  | 138 | ± | 10 | 84 |
| - C^b^ | 434 | ± | 36 |  | 423 | ± | 26 | 97 |
| - PD^b^ | 557 | ± | 59 |  | 418 | ± | 38* | 75 |
| - Input^b^ | 546 | ± | 67 |  | 461 | ± | 41 | 84 |
| - WU^b^ | 445 | ± | 65 |  | 409 | ± | 37 | 92 |
| Mechanical-evoked  responses (number of APs/sec) |  |  |  |  |  |  |  |  |
| - 8g | 16 | ± | 4 |  | 9 | ± | 1 | 56 |
| - 10g | 25 | ± | 5 |  | 14 | ± | 2 | 56 |
| - 15g | 47 | ± | 6 |  | 26 | ± | 3**^#^** | 55 |
| - 26g | 57 | ± | 6 |  | 42 | ± | 3 | 74 |
| Acetone -evoked responses (number of APs/sec) | 5 | ± | 0 |  | 6 | ± | 2 | 120 |
| Spontaneous activity (number of APs/sec) | 0.4 | ± | 0.13 |  | 0.5 | ± | 0.31 | 125 |

* p = 0.0475, unpaired t-test  ^#^p<0.0257, Mann-Whitney U test, compared to the control group

^a^Responses electrically following 3xAβ fibre threshold stimulation

^b^Responses electrically following 3xC-fibre threshold stimulation

APs; action potentials

**Table S4 Correlations between genes involved in the resolvin system and other selected genes studied**

|  |  | *Resolvin system* | | | | | | | | | |
| --- | --- | --- | --- | --- | --- | --- | --- | --- | --- | --- | --- |
|  |  | *Cyp2e1* | *Alox5* | *Cyp2j4** | *Alox5ap** | *Ptgr1** |  | *Fpr2* | *Cmklr1* | *Ltb4r* | *GPR18* |
|  |  | Resolvin degradation enzyme | Resolvin synthetic enzyme | Resolvin degradation enzyme (but generate other pronociceptive lipids) | Resolvin synthetic enzyme activating protein | Resolvin degradation enzyme |  | RvD1 Receptor | RvE1 receptor | RvE1 receptor | RvD2  receptor |
| *Tlr4* | Proinflammatory receptor |  |  |  |  |  |  | p=0.0402  r=-0.7761 |  | p= 0.0055  r= -0.9386 |  |
| *Cd14* | TLR4 co-receptor |  |  |  |  |  |  |  |  |  |  |
| *Otulin* | NF-kB signalling |  |  |  |  |  |  |  |  |  |  |
| *IL-8*  *(Cxcl8)* | Proinflammatory cytokine |  | p= 0.0017  r= 0.9659 |  |  |  |  |  | p= 0.0008  r= 0.9921 |  | p= 0.0154  r=- 0.945 |
| *Il6r* | Proinflammatory cytokine receptor |  |  |  |  |  |  |  |  |  |  |
| *Tnfrsf1a** | Proinflammatory receptor |  |  |  |  |  |  |  |  |  |  |
| *Tnfrsf1b** | Proinflammatory receptor |  |  |  |  |  |  |  |  |  |  |
| *Myd88* | Proinflammatory signalling protein |  |  |  |  |  |  |  |  |  |  |
| *Nlrp1a** | Proinflammatory signalling protein |  |  |  |  |  |  |  |  |  | p=0.0467  r=-0.8838 |
| *Cxcl6** | Chemokine |  |  |  |  |  |  |  |  |  |  |
| *Ccr1** | Chemokine receptors |  |  |  |  |  |  |  |  |  |  |
| *Ccr2* | Chemokine receptors |  |  |  |  |  |  | p= 0.0060  r=0.8984 |  |  |  |
| *Ccr3* | Chemokine receptors |  |  |  |  |  |  |  |  | p= 0.0463  r=0.8845 |  |
| *Ccr7* | Chemokine receptors |  |  |  | p= 0.0493  r= 0.8128 | p= 0.0341  r= 0.8452 |  |  |  | p= 0.0162  r=0.943 |  |
| *Cxcr5** | Chemokine receptors |  |  |  |  |  |  |  |  |  |  |
| *Cxcr1** | Chemokine receptors |  |  |  |  |  |  |  |  |  |  |
| *Cx3cr1** | Chemokine receptors |  |  |  |  |  |  |  |  |  |  |
| *Socs2** | Antiinflammatory signalling protein |  |  |  |  |  |  |  |  |  |  |
| *Pparg** | Antiinflammatory signalling protein |  |  |  |  |  |  |  |  |  |  |
| *Grin2B** | Glutamate NMDA receptor subunit |  |  |  |  |  |  |  |  |  |  |
| *Casp1^IPA^* | Cleaves ProIL-1β to active IL-1β |  | p= 0.0075  r= 0.9659 |  | p= 0.0400  r= 0.8954 | p= 0.0060  r= 0.9707 |  |  |  |  |  |
| *Gria1 ^IPA^* | Glutamate AMPA receptor subunit |  |  |  |  |  |  |  |  |  |  |
| *GFAP ^IPA^* | Astrocyte activation |  |  |  | p= 0.0242  r= 0.8702 | p= 0.0024  r= 0.709 |  |  |  |  |  |
| *Aif1* | Microglia activation (IBa1) |  |  |  | p= 0.0097  r= 0.9186 |  |  |  |  |  |  |

* = identified as significant genes by the 2^-ΔΔCt^ method,^IPA^= identified as significant genes by IPA analysis

**Figure S1 Behavioural pain responses in the PCX model and carrageenan model**

(**A** Paw withdrawal thresholds (PWTs) were measured at baseline and following induction of the PCX model (n=7) or vehicle (n=7) for up to 28 days. (**B**) Weight bearing difference and (**C**) paw withdrawal thresholds (PWTs) following intraplantar injection of carrageenan (n=7) or saline (n=6) for up to 24 hours. Analysis: two-way ANOVA with Sidak post hoc tests: **p<0.01, ***p<0.001, ****p<0.0001.

**Figure S2 mRNA expression profile in rat ipsilateral dorsal horn of the spinal cord in the PCX and carrageenan models.**

Data are fold change relative to the respective control. Analysis: Mann-Whitney U Test comparing raw C_T_ values of each treatment versus its control^, #^p<0.05, ^##^p<0.01 (n=5-7/group).

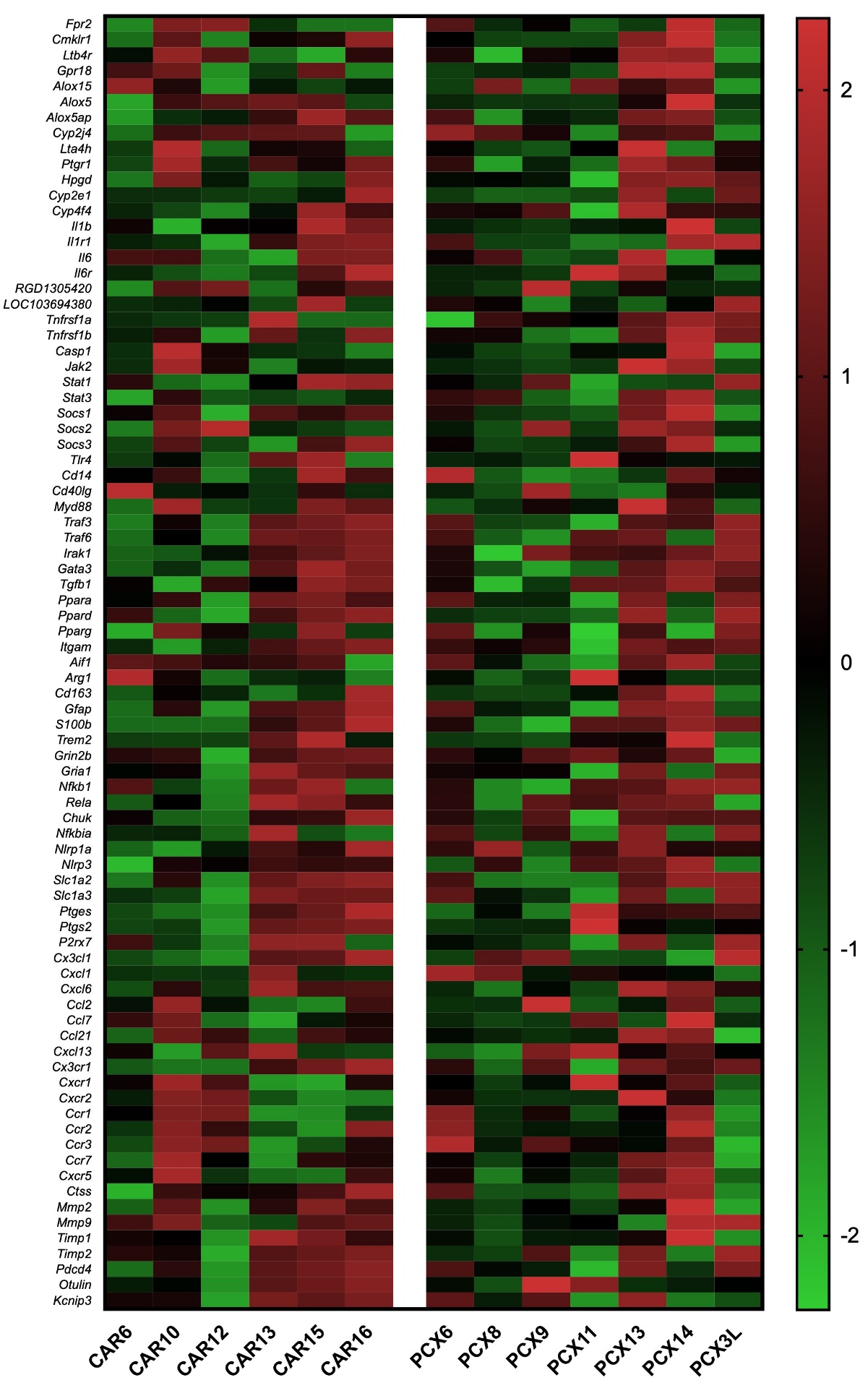
**Figure S3** Heat map of individual mRNA abundance relative to appropriate control (saline) in the ipsilateral dorsal horn of the spinal cord of rats following induction of the inflammatory pain model (carrageenan, n=6) and the model of chemotherapy induced neuropathic pain (PCX n= 7). Red signifies greater relative abundance, while green signifies less relative abundance.

**Figure S4** Correlations between pain behavior (number of von Frey filament changed from baseline (ΔvF)) and expression levels of genes involving generation and catabolism of the resolvin molecules.

**Figure S5** The synthetic and catabolic pathways for the resolvins


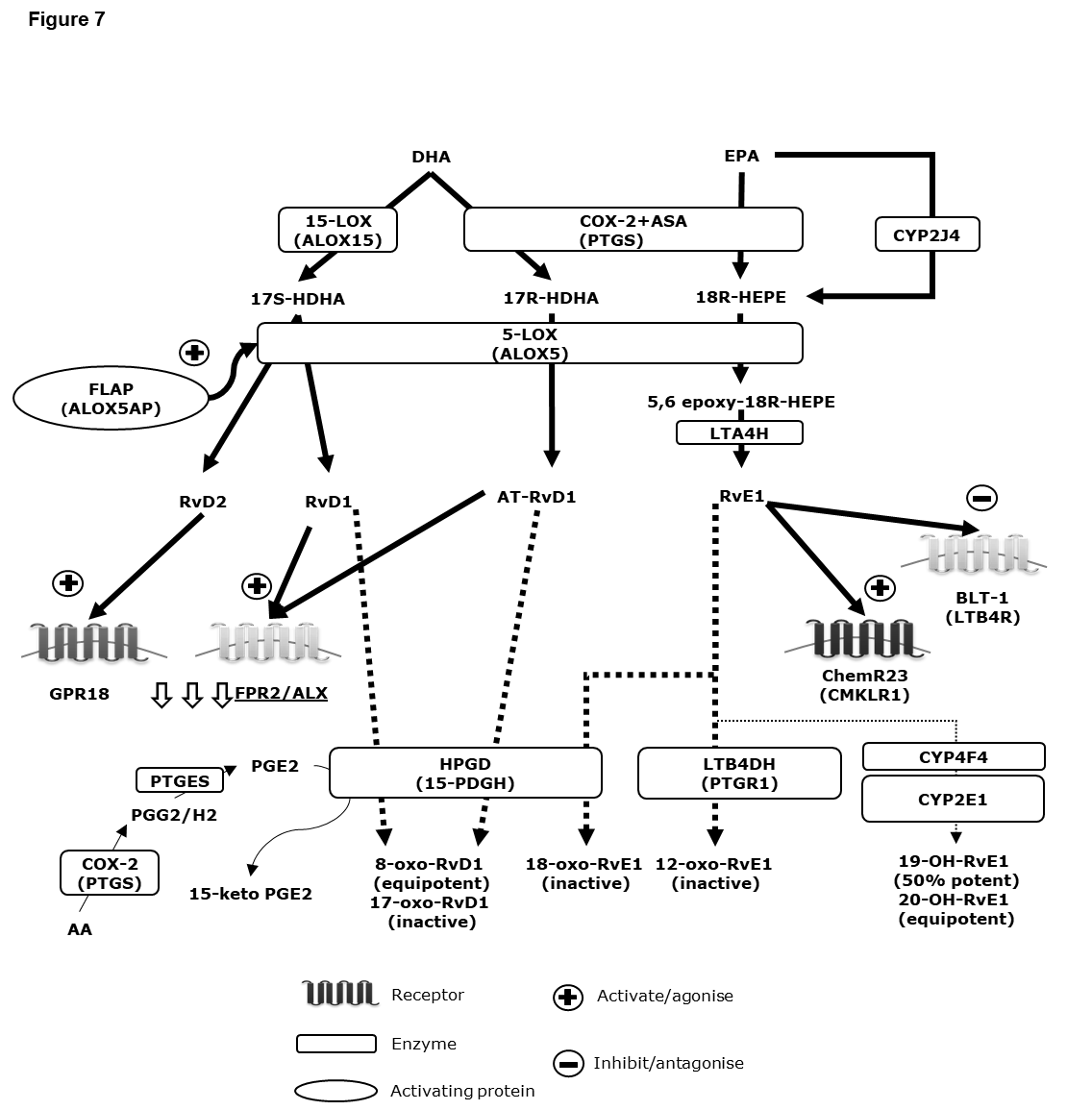


**Abbreviations**

**Precursor and metabolite** 17-R-HDHA: 17-R-hydroperoxy docosahexaenoic acid, 17S-HDHA: 17S-hydroperoxy docosahexaenoic acid, 18-oxo-RvE1: 18-oxo-RvE1, 19-OH-RvE1: 19- or 20-hydroxy-RvE1: 19- or 20-hydroxy-resolvin E1, 5,6-epoxy-18R-HEPE: 5,6-epoxy,18R-hydroxy eicosapentaenoic acid, 8-oxo-RvD1: 8-oxo-resolvin D1, AA: arachidonic acid, ASA: acetyl salicylic acid or aspirin, AT-RvD1: aspirin-triggered resolvin D1, DHA: docosahexaenoic acid, EPA: eicosapentaenoic acid, LXA4: lipoxin A4, RvD1: resolvin D1, RvD2: resolvin D2, RvE1: resolvin E1

**Enzyme and activating protein** 15-LOX: 15-lipoxygenase, 5-LOX: 5-lipoxygense, COX-2:cyclooxygenase, CYP: cytochrome P450, FLAP: 5-LOX activating protein, HPGD: hydroxyprostaglandin dehydrogenase, LTA4H:leukotriene A4 hydrolase, LTB4DH: leukotriene B4 12-hydroxydehydrogenase, PTGS2: prostaglandin endoperoxide synthase 2,

**Receptor** BLT1: leukotriene B4 receptor1, ChemR23: chemerin receptor23, FPR2/ALX: formyl peptide receptor 2, GPR18: G protein-coupled receptor 18, LTB4: leukotriene B4
